# Supplementary material for: Endothelial Nitric Oxide Mediates the Anti-Atherosclerotic Action of Torenia concolor Lindley var. Formosama Yamazaki
Source: Int J Mol Sci. 2020 Feb 24;21(4):1532. doi: 10.3390/ijms21041532 (PMC7073175; doi:10.3390/ijms21041532)
Supplement: Supplementary file 1 [file ijms-21-01532-s001.pdf]

Supplementary Material

Endothelial Nitric Oxide Mediates the Anti-Atherosclerotic Action of *Torenia Concolor* Lindley var. *Formosama* Yamazaki

Li-Ching Cheng, Bei-Chia Guo, Chia-Hui Chen, Chi-Jen Chang, Ta-Sen Yeh, Tzong-Shyuan Lee

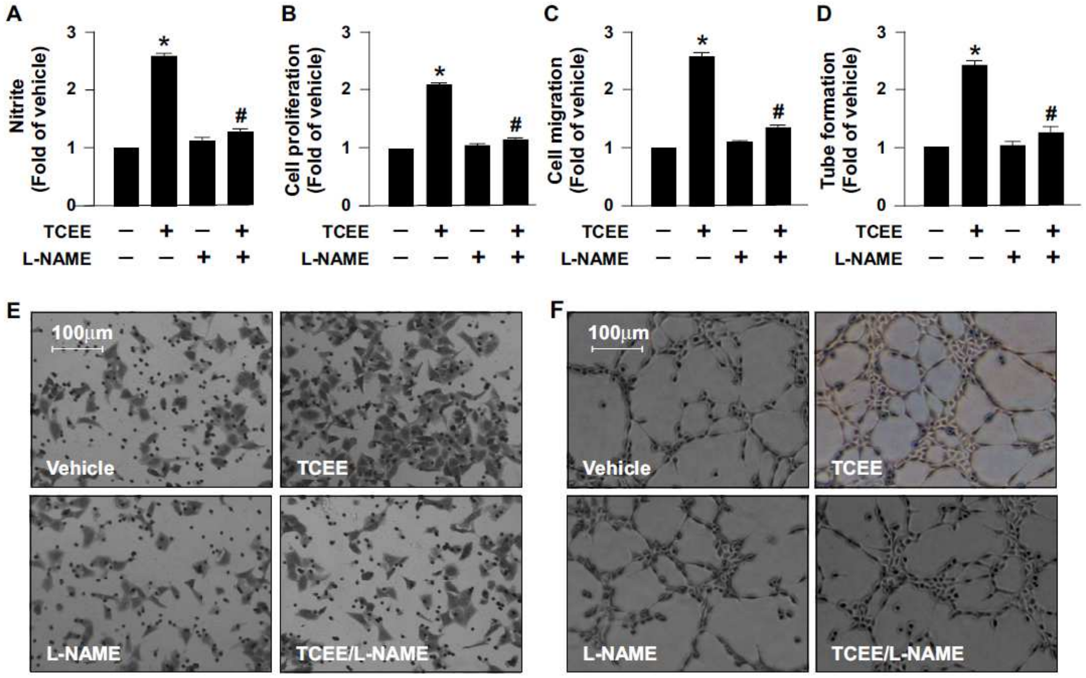

Figure S1. **The role of eNOS-derived NO in the TCEE-mediated beneficial effects on physiological functions of human microvascular EC (HMECs).** HMECs were pretreated with L-NAME (400 μM) for 1 h, then with TCEE (2 μg/ml). (A) The level of nitrite in culture medium, (B) EC proliferation, (C and E) EC migration and (D and F) tube formation were evaluated. The images were generated by microscopy. Data are mean ± SEM from 5 independent experiments. *P< 0.05 vs. vehicle-treated group; $^{\#}$P< 0.05 vs. TCEE-treated group.

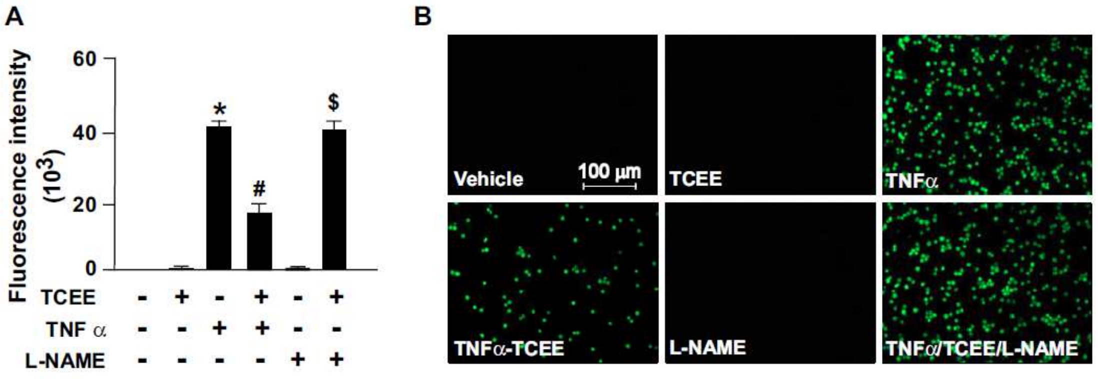

Figure S2. **TCEE inhibits the TNFα-induced adhesion of monocytes onto ECs in human microvascular ECs (HMECs)**. HMECs were pretreated with TNFα (10 ng/ml) or the vehicle for 12 h, followed by the treatment with L-NAME (400 μM) for 1 h and/or TCEE (2 μg/ml) for another 18 h. (A and B) BCECF-AM-labeled human monocyte THP-1 cells ($1x10^5$) were added and incubation with ECs for 1 h. The cellular lysates were subjected to fluorometry and photo-micrographed. Data are mean ± SEM from 5 independent experiments. *$P< 0.05$ vs. vehicle group; $^{\#}P< 0.05$ vs. TCEE-treated group; $^{\$}P< 0.05$ vs. TNF-α+TCEE-treated group.
